# Challenges for Ex Situ Conservation of Wild Bananas: Seeds Collected in Papua New Guinea Have Variable Levels of Desiccation Tolerance

**DOI:** 10.3390/plants9091243

**Published:** 2020-09-21

**Authors:** Simon Kallow, Kevin Longin, Natalia Fanega Sleziak, Steven B. Janssens, Filip Vandelook, John Dickie, Rony Swennen, Janet Paofa, Sebastien Carpentier, Bart Panis

**Affiliations:** 1 Royal Botanic Gardens Kew, Millennium Seed Bank, Wakehurst, Ardingly, Sussex RH17 6TN, UK; j.dickie@kew.org; 2 Department of Biosystems, Katholieke Universiteit Leuven, Willem de Croylaan 42, 3001 Leuven, Belgium; kevin.longin@kuleuven.be (K.L.); rony.swennen@kuleuven.be (R.S.); sebastien.carpentier@kuleuven.be (S.C.); bart.panis@kuleuven.be (B.P.); 3 Meise Botanic Garden, Nieuwelaan 38, 1860 Meise, Belgium; steven.janssens@plantentuinmeise.be (S.B.J.); filip.vandelook@plantentuinmeise.be (F.V.); 4 Bioversity International, Willem de Croylaan 42, 3001 Leuven, Belgium; natalia.fanegasleziak@kuleuven.be; 5 Plant Conservation and Population Biology, Katholieke Universiteit Leuven, Kasteelpark Arenberg 31, 3001 Leuven, Belgium; 6 International Institute of Tropical Agriculture, c/o Nelson Mandela African Institution of Science and Technology, Arusha 0206, Tanzania; 7 National Agricultural Research Institute, Laloki 121, Papua New Guinea; janet.paofa@nari.org.pg

**Keywords:** banana, crop wild relatives, ex situ conservation, desiccation tolerance, *Musa*, Papua New Guinea, plant genetic resources, seed conservation, seed storage behaviour

## Abstract

Ex situ seed conservation of banana crop wild relatives (*Musa* spp. L.), is constrained by critical knowledge gaps in their storage and germination behaviour. Additionally, challenges in collecting seeds from wild populations impact the quality of seed collections. It is, therefore, crucial to evaluate the viability of seeds from such collecting missions in order to improve the value of future seed collections. We evaluate the seed viability of 37 accessions of seven *Musa* species, collected from wild populations in Papua New Guinea, during two collecting missions. Seeds from one mission had already been stored in conventional storage (dried for four months at 15% relative humidity, 20 °C and stored for two months at 15% relative humdity, −20 °C), so a post-storage test was carried out. Seeds from the second mission were assessed freshly extracted and following desiccation. We used embryo rescue techniques to overcome the barrier of germinating in vivo *Musa* seeds. Seeds from the first mission had low viability (19 ± 27% mean and standard deviation) after storage for two months at 15% relative humidity and −20 °C. *Musa balbisiana* Colla seeds had significantly higher post-storage germination than other species (*p* < 0.01). Desiccation reduced germination of the seeds from the second collecting mission, from 84 ± 22% (at 16.7 ± 2.4% moisture content) to 36 ± 30% (at 2.4 ± 0.8% moisture content). There was considerable variation between and (to a lesser extent) within accessions, a proportion of individual seeds of all but one species (*Musa ingens* N.W.Simmonds) survived desiccation and sub-zero temperature storage. We identified that seeds from the basal end of the infructescence were less likely to be viable after storage (*p* < 0.001); and made morphological observations that identify seeds and infructescences with higher viability in relation to their developmental maturity. We highlight the need for research into seed eco-physiology of crop wild relatives in order to improve future collecting missions.

## 1. Introduction

Crop wild relatives (CWRs) possess genetic material useful for improving crops in an increasingly challenging context [1,2,3]. They comprise a large untapped genepool of alleles potentially useful for breeding [4]. Examples from banana CWRs include improved drought tolerance [5] and resistance to several diseases [6,7]. At the same time, many CWRs are threatened with extinction [8,9] making their conservation imperative for both biodiversity and food security [10,11]. Effective plant conservation employs complementary in situ and ex situ strategies [12,13]. Such an approach is notably encouraged for CWR conservation [14,15]. Accordingly, ambitious efforts to collect and conserve many CWRs ex situ have recently been made [16,17,18,19,20]. However, CWRs, and banana CWRs (*Musa* spp. L.) in particular, are poorly represented in ex situ collections [21]. 

Banana CWRs are diploid wild species whose fruits contain many dark coloured seeds. Edible bananas, selected to avoid seeds in the fruit pulp, can be diploid, triploid or tetraploid. There are around 80 species in the genus *Musa* [22,23], and over 1000 edible banana cultivars [24,25]. The management of banana germplasm is co-ordinated in a global network of 31 collections containing over 6600 accessions of in vitro or field plants [26]. Only 163 of these accessions are CWRs. Moreover, of these, 122 are of the two most important banana CWRs (*Musa acuminata* Colla and *M. balbisiana* Colla), the other 41 accessions include 33 *Musa* species. Additionally, there are 131 *Musa* seed collections of only seven species stored in seed banks [27]. This means many species are only represented by a single genotype and for many wild banana species, no accessions exist. The diversity of banana CWRs in ex situ conservation is therefore highly constrained and expansion of the inter and intra-specific diversity of the collection is clearly needed. 

*Musa* are pioneers or early successional tall herbs of tropical to subtropical rainforests. Native distribution area ranges from Southeast Asia to Pacific regions [22]. Papua New Guinea (PNG), the world’s most floristically diverse island [28], is an important centre for both wild banana and cultivar diversity [29,30,31,32]. Sixteen wild *Musa* taxa occur in PNG [33]. Several collecting missions have been made in PNG to characterise and collect both cultivar and CWR germplasm [34,35,36,37,38]. These include seed collections, two of which we evaluate here.

Ex situ conservation using seeds can be a highly effective way of conserving the genetic diversity of plant populations [39,40]. This is useful for further conservation activities, phenotyping and breeding. Furthermore, conservation using seed is a relatively cost effective method of ex situ conservation [41]. In order to make high quality seed collections of wild species, understanding of seed development and storage behaviour are crucial [42]. Seed storage behaviour can classically be categorised into three groups. The majority of seeds are easily dried (to 2–5% MC) and stored at sub-zero temperatures, these are *orthodox* seeds [43]. Secondly, *recalcitrant* seeds do not survive drying to below 20–30% moisture content and are sensitive to low temperatures [43]. Finally, seeds that do not fit well into these binary categories are often called *intermediate,* and show partial sensitivity to drying and cold storage in particular [44]. Seeds of recalcitrant and intermediate species should be stored cryogenically, whereas orthodox seeds may be stored conventionally (at −20 °C) following desiccation [45].

For wild species, and especially banana CWRs, critical knowledge gaps exist in how best to collect, store and germinate their seeds. For *Musa*, only six species have been assessed for their storage behaviour, results of which are inconclusive [46,47,48,49,50,51,52]. Additionally, germination of seeds is notoriously inconsistent and dormancy poorly understood [53,54,55]. Embryo rescue techniques are therefore commonly used to germinate seeds in breeding programmes [56]. Together, these critical knowledge gaps hamper storage and access to banana genetic material [54]. 

Substantial challenges associated with collecting seeds from wild species impact the quality of seed collections [57]. Non-uniform seed development across a population, low seed numbers and sub-optimal post-harvest handling may be problematic [57,58,59,60]. Post-harvest handling is difficult because it is often not possible to control the temperature and humidity of seeds on collecting missions, e.g., whilst in a vehicle or when moving from place to place. Furthermore, there are significant practical challenges in collecting seed material from populations of wild species, the location of which may be remote, inaccessible and previously unknown. Evaluation of material from actual collecting missions can provide useful concrete evidence of these particular challenges, and lessons can be learnt to improve the quality of collections in the future. 

In this study, we make use of seeds from two recent collecting missions to PNG. Seeds from one mission were already stored in Meise Botanic Gardens seed bank (called ‘batch 1’, and described by Eyland et al. [38]); the others were collected during the course of this investigation (called ‘batch 2’). By evaluating seed viability of these collections, we address some of the issues and knowledge gaps described, by answering the following questions: (1) What is the viability of *Musa* seeds stored in Meise Botanic Gardens seed bank (for two months at 15% relative humidity (RH), −20 °C)? (2) Do seeds of some *Musa* species have higher viability after storage than others? (3) Do seeds from different parts of the infructescence have higher viability after storage than others? (4) How does desiccation affect seed viability? (5) Is it possible to predict storage behaviour of *Musa* seeds based on their physical properties? (6) Does seed maturity affect viability during dry storage? We use in vitro embryo rescue techniques to quantify viability. This provides the most reliable estimate of viability and removes dormancy constrains that limit germination in *Musa* seeds [61,62,63].

## 2. Results

### 2.1. Viability Evaluation of Seeds Stored in the Seed Bank

#### 2.1.1. Overall Viability

**Figure 1:**
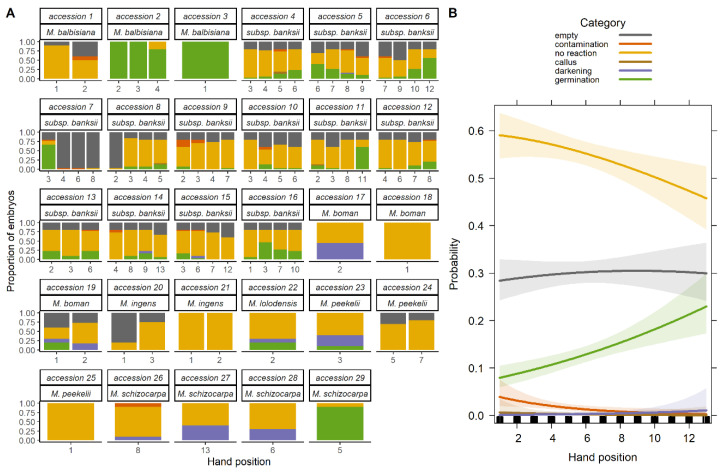
(**A**) Germination responses of embryos rescued from 29 accessions of *Musa* species following drying for 4 months at 15% relative humidity 20 °C and storage for 2 months at 15% relative humidity −20 °C. ‘Hand position’ refers to the position in the infructescence of the hand from which seeds were collected, with ‘1’ being closest to the basal end of the bunch (*n* = 23 ± 10 seeds). (**B**) Predicted probability of five embryo rescue outcomes of *Musa acuminata* subsp. *banksii* seeds extracted from different hand positions in the infructescence. Probabilities based on the multinomial logistic regression model of the response of seeds from 50 hands (representing 13 accessions; *n* = 30 seeds for each hand). Shaded areas are 95% standard errors of the estimated regression coefficients.

The post-storage viability of batch 1 seeds (already stored in Meise Botanic Gardens seed bank) was markedly low with considerable variance between the accessions (Figure 1A). Across all accessions and hands, germination was on average 19 ± 27% (mean and standard deviation used hereafter; empty seeds are excluded in the percentages). In the present study, we use the term *accession* to mean a seed collection from a single individual plant including all the fruits of an infructescence. The term *bunch* refers to an infructescence. A bunch can be subdivided into *hands,* these are groups of fruits from the former clusters of flowers subtended by one bract [64]. Embryos that showed no reaction were 73 ± 29%. Other embryonic reactions, callus formation and embryo darkening without further outgrowth, were minimal (respectively, 0.2 ± 1.2%, and 3 ± 9%). Microbial contamination of the sample was 4 ± 16%. Overall, 24 ± 23% of seeds contained no identifiable embryos. 

#### 2.1.2. Effect of Species

*Musa balbisiana* Colla seeds (accessions #2 and #3), showed significantly higher germination than other species after storage (*p* < 0.01), in parallel with less embryos showing no reaction. This is demonstrated by the multinomial logistic regression (MLR) model (Table S1A), whereby the log odds of germination against an increase in no reaction is 0.865, but for all other species, log odds are negative. This is despite one of the three *M. balbisiana* accessions (accession #1) having no viability. One *M. schizocarpa* accession also showed high viability (accession #29, 90%), in contrast to the other three (accessions #26–28).

#### 2.1.3. Effect of Position in the Infructescence

*Musa acuminata* subsp. *banksii* seeds from hands with a higher number, i.e., that were more recently pollinated, had embryos that were significantly more likely to germinate after storage (*p* < 0.001, Figure 1B, Table S1B); again this was concurrent with a reduced likelihood of no reaction. 

### 2.2. Effect of Desiccation

#### 2.2.1. Overall Effect of Desiccation

**Figure 2:**
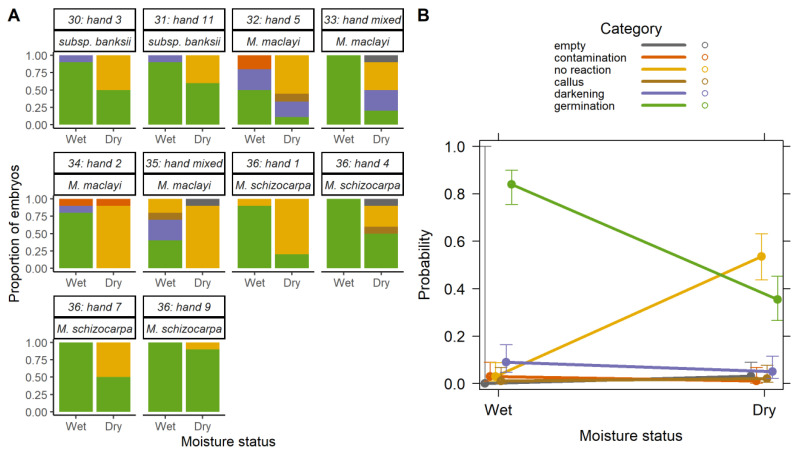
(**A**) Embryo rescue outcomes of *Musa* seeds (batch 2) before desiccation at 16.7 ± 2.4% moisture content (‘Wet’), and after desiccation for seven days in a desiccator to 2.4 ± 0.8% moisture content (‘Dry’). Accession and hand numbers are included above each chart. Seeds were germinated using embryo rescue and results recorded 28 days after transfer to the growth medium (*n* = 10). (**B**) Predicted probability of embryo rescue results according to the moisture status of seeds. Plot is on predicted values of the multinomial logistic regression model coefficients in Table S1C, data in Figure 2A. 95% standard errors shown.

Seeds of batch 2 were tested before and after desiccation. Before desiccation (16.7 ± 2.4% moisture content (MC)), germination was on average 84 ± 22% (Figure 2A). After seven days desiccation (to 2.4 ± 0.8% MC), germination decreased to 36 ± 30%. As embryos dried, their percentage without any sign of germination increased from 3 ± 7% to 55 ± 27%. Again, there was considerable variance between accessions. Accession #37, *M. balbisiana*, was excluded from the analysis as the initial MC was an outlier compared to all the others (35% MC).

#### 2.2.2. Desiccation Tolerance and Species

Viability after desiccation differed by species. The *M. acuminata* subsp. *banksii* and *M. schizocarpa* accessions showed the highest germination after desiccation (55 ± 7%, 54 ± 29%, respectively). *M. maclayi* accessions had the lowest viability after desiccation (3 ± 6%). The MLR model based on drying as a factor showed a significant effect on germination in relation to no reaction (*p* < 0.001, Figure 2B, Table S1C). Additionally, seeds in wet condition were also more likely to darken and be contaminated (*p* < 0.001, *p* < 0.01, respectively). In the model, there is clear interchange of embryos that germinate with those that show no reaction (Figure 2B).

### 2.3. Prediction of Seed Storage Behaviour

**Figure 3:**
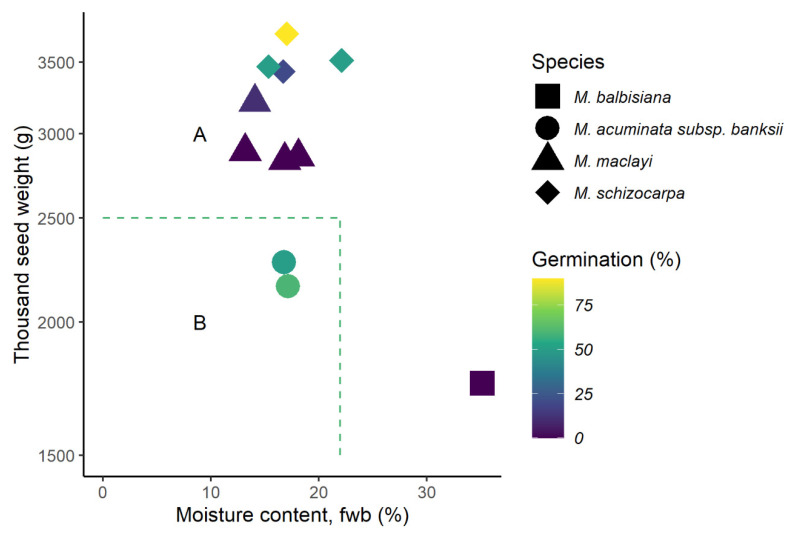
Predicted storage of behaviour of *Musa* accessions (batch 2) using the diagnostic key of Hong and Ellis [65] and Ellis et al. [66]. Area A includes accessions predicted to have intermediate storage behaviour, accessions in area B are predicted to have orthodox storage behaviour. Accessions are coloured according to the germination percentage of seeds after seven days desiccation to 2.4 ± 0.8% moisture content. Moisture content is calculated on the fresh weight basis (‘fwb’). Seeds were germinated using embryo rescues and assessed 28 days after transfer to growth medium.

Predicted seed storage behaviour (using the method of Hong and Ellis [65] and Ellis et al. [66]), identified that accessions straddled both intermediate and orthodox categories (Figure 3). Nine of the 11 accessions were predicted to be intermediate and two orthodox. The accessions predicted to be intermediate exceeded the threshold for weight rather than moisture content (apart from accession #37, *M. balbisiana,* previously identified as an outlier with high MC). There was no correlation between predicted seed storage behaviour and post-desiccation germination. Only seeds from batch 2 were used, as moisture content measurement of seeds before desiccation is required by the model. 

### 2.4. Dry storage and Maturity

#### 2.4.1. Effect on Viability 

**Figure 4:**
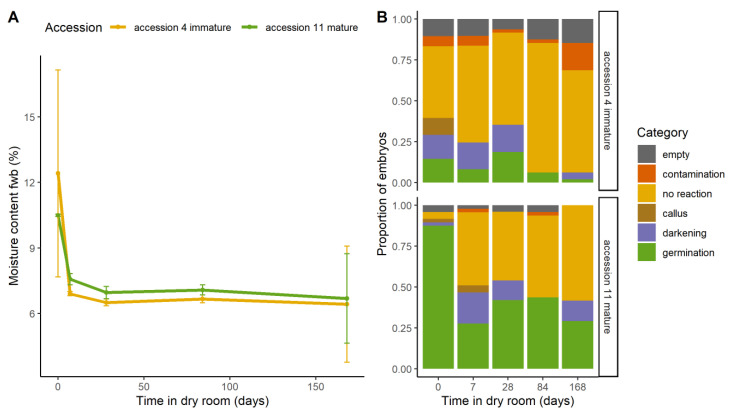
(**A**) Moisture content during dry storage, calculated on fresh weight basis (‘fwb’). Seeds from a mature and an immature accession of *Musa acuminata* subsp. *banksii* were used. Seeds were dry stored at 15%RH, 20 °C for up to 168 days. (**B**) The effect of dry storage on embryo rescue outcomes. Outcomes recorded 28 days after the transfer of each embryo to growth medium (*n* = 48).

Two *M. acuminata* subsp. *banksii* accessions were selected from batch 1. Accession #4 was from a less mature and accession #11 from a more mature bunch according to observations in the field. Seeds were tested before and during dry storage (contained within a paper bag, stored in a humidity controlled room at 15%RH, 20 °C). Seeds had a mean moisture content of 11.2 ± 2.7% before drying (Figure 4A). After seven days drying, moisture content reduced to 6.6 ± 2.5%. Moisture content remained about the same with further drying time, so that after six months moisture content was 6.5 ± 2.35%. Differences in moisture content between the accessions were not statistically significant.

The mature seeds (accession #11) had much higher initial germination rates than the immature (accession #4), 91.3% and 16.3%, respectively (Figure 4B). Embryos from both accessions reduced in germination after seven days drying, to 28.3% for the mature accession and to 9.1% for the immature accession. For the mature seeds, this level of germination remained about the same with further drying time, so that after 6 months of drying, germination was 29.2%. Embryos from the immature accession continued to reduce in germination with further drying time, so that after 6 months dry storage, germination was negligible (2.4%). The proportion of embryos that germinated was notably reduced and exchanged to a correspondingly larger proportion of embryos that displayed no germination reaction, and to a lesser extent, darkening. Contamination also increased with time of drying for the immature seeds.

#### 2.4.2. Effect on Morphology

**Figure 5:**
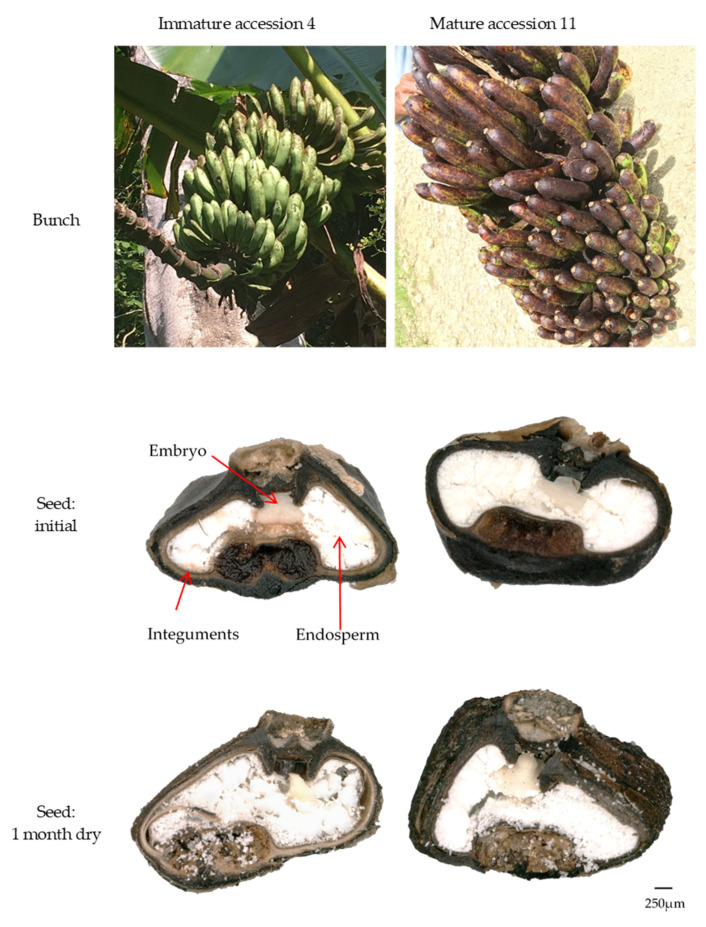
Photographs of immature and mature bunches and their seeds of two *Musa acuminata* subsp. *banksii* accessions. Seed images taken before and after 1 month drying in a dry room (15% relative humidity, 20 °C). Seed images taken on a Keyence VHX5000 at 150 x magnification.

Observations from magnified images of the selected *M. acuminata* subsp. *banksii* accessions showed apparent under-developed seed coats in the less mature seeds. The different layers of the seed-coat integuments are evident rather than fused, they are also lighter in colour (Figure 5). During drying, these layers were observed to separate. Additionally, there is a noticeably greater effect of desiccation on the structure of the endosperm and shape of the embryo. Less mature seeds display increased airspaces in the endosperm on desiccation. Embryos of the less mature seeds show greater loss of structure during desiccation. 

## 3. Discussion

### 3.1. Key Findings

This assessment of seed storability of banana CWR seeds from PNG collecting missions illustrates some of the challenges involved in making high quality collections of wild species for ex situ conservation. In particular, this assessment demonstrates some of the difficulties involved in making seed collections of wild species and how critical knowledge gaps impact the value of such collections. Our evaluation shows substantial loss of seed viability during seed banking which can be attributed to variable levels of desiccation tolerance. There was considerable variation between accessions, and some species (*M. balbisiana* and to a lesser extent *M. schizocarpa* and *M. acuminata* subsp. *banksii*) maintained higher viability during storage compared to others.

### 3.2. Desiccation Sensitivity

The low germination rates (19%) of seeds that were stored in the seed bank (batch 1), suggests a problem with maintaining the viability of collections in conventional storage (15%RH, −20 °C). However, as this was a viability assessment of seeds already stored, it is not possible to draw specific conclusions as to why viability is low: seeds may have had low initial viability or lost viability during transport, for example. By testing batch 2 seeds both before and after desiccation, it is clear that desiccation sensitivity is a major contributor to loss in viability. On average, these seeds reduced germination from 84% to 36% during rapid desiccation (from 17% to 2.4% moisture content). We therefore surmise that loss of viability is primarily a result of sensitivity to rapid desiccation. Further research is needed to fully understand whether loss in viability is caused by desiccation per se, or whether speed of desiccation is an important contributing factor. 

### 3.3. Seed Storage Behaviour

Variation in our results, with respect to seed storage classification, is in line with previous studies. For instance, several studies demonstrate desiccation sensitivity where seeds lose viability at 6% MC [46] or, for extracted embryos, to 10–15%MC [50,51,67]. Other studies found that seeds tolerate drying, but do not state to what moisture content [47,48]. 

High viability of a few accessions stored under very low moisture and sub-zero temperature in the present study, suggests that (at least for *M. balbisiana*) orthodox storage class is likely. For others, our results suggest that intermediate storage classification may be appropriate, as significant proportions of seeds lost viability on desiccation and freezing. *Musa* storage behaviour is, therefore, at the threshold between orthodox and intermediate storage classes, as illustrated by results of the predictive model (Figure 3). It should be noted, however, that for all species (apart from *M. ingens*), a proportion of seeds survived desiccation, or even desiccation and sub-zero storage. Storage class was, therefore, variable within an accession, and orthodox behaviour of at least a small proportion of seeds was possible, if rare. Whilst storage classification is helpful, a continuum of storage behaviour is known to exist [68], even within the same genus or species, depending on when and where seeds were collected [45,69,70,71,72]. In the present study, a continuation in desiccation tolerance is evident within the same accession and even from fruits in the same hand. 

### 3.4. Variation between Infructescences

#### 3.4.1. Species and Climate

Differences in post-storage viability were greater between-infructescences (from different maternal plants), than within-infructescences (from hands of the same plant, Figure 1A). This may be related to differences in seed storage behaviour at the species level, to the maturity level of the whole infructescence or perhaps the microclimate [73]. Viability levels were consequently strongly linked to the fruit-bearing plant. 

In batch 1, *M. balbisiana* seeds showed significantly higher post-storage viability than the other species. This species is characterized by a wide, yet often introduced, distribution across the tropics and subtropics. Notably, *M. balbisiana* is not considered a native species to PNG [74,75], but rather has its native distribution in the more seasonal subtropical Northern Indo-Burmese region [76]. By contrast, the other wild banana species studied are native to the Equatorial wet to moist ecoregions of PNG. As such, *M. balbisiana*, has also shown to have high leaf wax content that contributes to drought tolerance [5] and is therefore probably better adapted to seasonal changes in precipitation and temperature than the *Musa* species native to PNG [33,74,75]. This then suggests that within the whole genus, there may well be a range of desiccation tolerance levels possibly according to species distributions. It should, however, be caveated that our observation is based on only a small number of samples and a wider survey should be carried out for further conclusions. Nonetheless, it is well known that there is a correlation between the bioclimatic distribution of a plant and seed storage behaviour: higher annual precipitation is positively correlated with recalcitrance [77]. Interestingly, differences in precipitation in the native region of the *Musa* species examined here are in fact greater when only the precipitation in the driest quarter of the year is considered, rather than for annual precipitation (see Figure S1). We therefore suggest that the precipitation in the driest quarter might possibly have a stronger correlation to seed storage behaviour for *Musa* than annual precipitation, as the possible impact of a dry season may be masked in seemingly high precipitation regions. We therefore propose that *Musa* seeds collected from species adapted to more pronounced dry seasons may have better desiccation tolerance and therefore better survive storage. However, further research is required in this area. 

#### 3.4.2. Seed Maturity

We identified physical properties that were seemingly linked to the level of seed maturity at the time of harvest. Larger fruits with softer pulp texture and seeds with a more powdery endosperm were considered to be more mature. Seeds from the bunch categorised as more mature in the field had greater embryo rescue germination percentages, both before and after dry storage (15%RH, 20 °C), compared to the seeds identified from the less mature bunch. In the laboratory, it was observed that the less mature seeds had higher initial moisture content that reduced to a greater extent, and an under-developed seed coat: a light brown inner integument as opposed to dark brown to black, that was less well fused with the outer integument (Figure 5). The small sample size notwithstanding, the importance of seed maturity for desiccation tolerance is consistent with current understanding of the development of desiccation tolerance during late seed maturation [73,78,79]. Desiccation tolerance is acquired at ‘mass maturity’ after maximum dry weight is achieved and the vascular connection between the maternal plant and seed is terminated [80]. Following this, seed moisture content equilibrates with the environment prior to dispersal. Often this is described as the ‘point of natural dispersal’ [60]. The difficulty for improving the quality of seed collections is how to translate theory into practice, particularly for seeds that are contained within large pulpy fruits like bananas. 

Regarding seeds that were collected from field collections, Simmonds [47] found that, for maximum in vivo germination, *M. balbisiana* seeds should be collected ‘mature’. Unfortunately, he did not define what ‘mature’ meant in this instance. However, he detected a window of six weeks whereby high germination can be achieved (>80%), four weeks before and two weeks after maturity. Additionally, he found that fruit of *M. acuminata* should be collected green or yellow (rather than black or rotten) to achieve high germination. Furthermore, Uma et al. [81] found that at 70% maturity (full maturity being 110 days after (self-)pollination) ‘Pisang Jajee’ (a *M. acuminata* genotype) embryos were discernible and endosperms had converted from a liquid to semi-solid state; this also coincided with thickening of the integuments. They also found that seeds, in order to germinate, should be at least 90% mature, and immature embryos were more likely to produce calluses. 

Collecting mature seeds during collecting missions is much more challenging than from field germplasm collections. Collectors must access bunches before they are consumed and seeds are dispersed by birds and mammals [82,83,84]. Humans also harvest wild bananas for food, construction and artistry [37,85]. It is therefore important to be able to identify fruits that contain seeds that are mature enough to be desiccation tolerant, without knowing flowering times, whilst they may not have yet attracted frugivores. Based on our results, we suggest that seeds should have powdery endosperms and well-formed integuments with fused layers, without which many seeds will be lost during storage; however, clearer definitions should be developed for collectors. 

It may also be possible to improve desiccation tolerance and longevity of seeds by using a treatment that mimics late maturation on the plant, as has been shown for other species [78,86,87,88]. Indeed, in one study [89], Simmonds found that seeds from ‘ripe’ and ‘over-ripe’ bananas that were dried in the fruit at a temperature similar to what may be found on the plant (in an oven at 45 °C), germinated better than seeds that were not dried in this manner. Assessing and furthering seed maturity whilst avoiding dispersal is clearly a key factor in improving the quality of future banana seed collecting. 

### 3.5. Variation within Infructescences

Heterogeneity of maturity within an infructescence has been highlighted as a cause for variable desiccation tolerance within seed accessions of other wild species [57,60]. We observed a small but significant within-infructescence effect, in that seeds from the male bud end of the infructescence (seeds from flowers that were more recently pollenated) were around 15% more likely to germinate (post-storage) than the peduncle basal end (Figure 1B). This, perhaps surprising, effect may be caused be caused by variation in seed-vigour or seed-aging, discussed below. 

It is well known for other species, that there are differences in physical and physiological properties in seed-vigour within the same infructescence [90]. For species of temperate regions variation is often correlated with seasonality [91,92,93]. In tropical species, the effect of climate on seed properties is not well known. However, pollen, seed set and germination success of banana seeds (during breeding programmes) have been found to correlate with climatic conditions [94,95]. Alternatively, seed-vigour, including the ability to tolerate stress, deteriorates according to temperature and moisture [71]. When seeds are kept in the fruit for relatively long periods of time, for example, during collecting missions, seed-aging can occur. This can potentially influence the ability of seeds to withstand the stress of desiccation later on. As seeds from basal hands are produced first, when they are harvested they are already in a more advanced state: fruit may soften quicker and have higher moisture content, and the exocarp may be rotting. This all means that aging is more likely to occur if they are then kept in the fruit during the remainder of the trip and until they are transported to the laboratory (see Figure S2 for a photograph of the fruits of batch 1 after transit to Belgium). This could explain why the older seeds within a bunch may display lower desiccation tolerance. However, as this effect was relatively small compared to the overall maternal effect, it seems that the within-bunch maturity appears at least to have less of an effect than the maturity of the whole bunch. 

### 3.6. Limitations and Assumptions

#### 3.6.1. Embryo Rescue

We used embryo rescue techniques to estimate viability in the present study. Whilst this is the best current method for estimating *Musa* seed viability, there are limitations and assumptions that should be stated. Firstly, the purpose of a viability measure is to estimate the proportion of seeds that are capable of developing into seedlings or plants [42]. Embryo rescue ‘short-cuts’ some of the constraints that could limit this process of in vivo germination. For instance, if an embryo germinates in vitro, it does not necessarily mean that it is capable of developing into a seedling or plant. For this to happen the embryo must also push off the seed micropyle cap and develop roots that can access the soil. We accounted for this in our analysis by categorising separately embryos that did not develop fully formed shoots, but rather formed calluses or showed no growth but darkening of the embryo. Secondly, embryo rescue evaluation, in our method, is at 28 days; however, it is possible that germination may be slow and only is evident after this period. According to the literature, 28 days should normally be enough time [61,62,96], but it is an assumption that, at this point in time, the germination process is concluded for all embryos. Finally, the conclusions of this study are based on the assumption that embryos showing no germination reaction are in fact dead. However, it is possible that desiccation does not kill the embryo, but rather causes a deep level of physiological dormancy that is not removed by excision from the rest of the seed. To account for this, we carried out tetrazolium tests on embryos that showed no germination reaction on embryo rescue. These embryos showed no staining. This indicates that embryo rescue produces the maximum measure of viability.

#### 3.6.2. Conservation and Research Material

Whilst the benefit of using seeds from collecting missions allows results to be impactful for future missions, limitations are also introduced by using such material. One of the main limitations we faced was the limited availability of seeds for research. This inevitably constrains the interpretation of results (hence the large amount of deviation) because sample sizes and replicates were small. Seed numbers were limited for two reasons. One, because it is difficult to access seed material in suitable time periods from third parties, despite relevant treaties [97]. Two, because there are conflicting demands for material. There is an expectation and requirement for seeds to be placed into storage ‘for conservation’. This may conflict with availability of adequate material for research into how best to store and germinate seeds. Our results highlight the need for seed collecting for research purposes in addition to, and ideally prior to, collecting missions whose primary purpose is conservation. In practice, as here, these two processes often run concurrently. 

## 4. Materials and Methods 

### 4.1. Study Region

**Figure 6:**
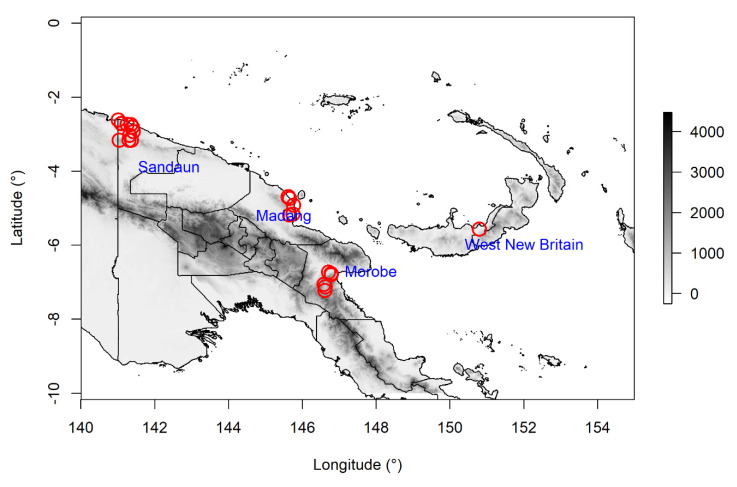
Collecting locations of seeds used in this study (red circles) and relevant province names. Map is shaded according to elevation (meters above sea level, data from: http://srtm.csi.cgiar.org).

The study region was between Latitude 2° to 8° South, and Longitude 141° to 151° East. Seeds were collected in the Papua New Guinean provinces of Morobe, Madang and Sandaun on the island of New Guinea, and the province of West New Britain on the island of New Britain (Figure 6). These locations are in the tropical and subtropical moist broadleaf forest biomes [98]. Mean annual precipitation and mean annual temperature, at the collecting locations are 2695 ± 562 mm and 24.9 ± 1.9 °C, respectively (averages for years 1970–2000) [99]. 

### 4.2. Plant Material

#### 4.2.1. Accessions

Overall, 37 *Musa* seed accessions were used in this study. Accessions were from a total of seven species: *Musa balbisiana* Colla*, M. acuminata* subsp. *banksii* (F. Muell.) N.W. Simmonds*, M. boman* Argent*, M. ingens* N.W. Simmonds*, M. lolodensis* Cheesman*, M. peekelii* Lauterb*, M. schizocarpa* N.W. Simmonds (Table 1).

#### 4.2.2. Seed Batches

Seeds were collected during two field missions to Papua New Guinea. Batch 1 was collected in May 2019, at the end of the wet season, and included 29 accessions, described by Eyland et al. [38]. Batch 2 was collected in October 2019, at the start of the wet season and contained eight accessions (Table 1). 

### 4.3. Seed Collection, Field Evaluation and Transportation

Seeds were collected from wild populations that occurred either in primary or secondary forests. At the time of collecting, seed maturity was assessed by dissecting approximately 10 seeds per bunch and examining the embryos and endosperms. Seeds were considered mature when embryos were capitate in shape (mushroom-like) and endosperms were powdery as opposed to wet or milky. Only bunches with seemingly mature seeds were collected (although, some bunches proved to be not completely mature, see results). Each bunch was photographed on site. Hands were removed from the bunch and numbered according to position, with 1 being at the basal end, i.e., they were produced first. Hands were placed in paper bags, which were then placed in cardboard boxes for storage during the remaining field mission. Accessions were then transported to Belgium for extraction. Transportation was initiated within one week of the end of the two-week collecting mission and took approximately one week to complete by aeroplane. During shipping, temperatures were greater than 0 °C and less than 25 °C. Fruits were therefore received within four weeks of collecting in the field.

### 4.4. Seed Processing

#### 4.4.1. Extraction

Seeds were extracted by peeling the epicarp and crumbling or squashing the endocarp and removing seeds by hand. Excess fruit pulp was removed by washing in running water if necessary. In case fruits were hard, they were soaked in water for 24 h prior to seed extraction. It took a week to extract all the seeds from batch 1, and one day for the seeds of batch 2. Seeds were maintained under ambient laboratory conditions (approximately 60–80% relative humidity, 20 °C) for a maximum of seven days whilst all extractions were completed, this also allowed removal of excess water gained during washing. Moisture content of a subset of three accessions of batch 1 seeds and all accessions of batch 2 seeds was measured after extractions were completed (see Section 4.4.2 for method). 

#### 4.4.2. Moisture Content Measurement

Moisture content (MC) was calculated on a fresh weight basis (FWB) using the formula:

$$MC\left(\%\right)=\frac{\left(fresh\;weight-dry\;weight\right)}{fresh\;weight}\;\times 100$$

Seeds were weighed in plastic boats, dried at 70 °C for three days, and re-weighed. The MC of seeds was then calculated. Seeds were dried whole, as seeds coats were previously assessed as water permeable (our own data not shown and see [100]). Our own previous results also showed that embryo moisture content was 2% higher than whole seeds for non-desiccated seeds (at 10%MC) and 3%MC higher after desiccation (to 3%MC). Whole seeds were used here because accurately measuring the moisture content of embryos requires many samples that were not available because of their small size. Three replicates of 10 seeds were used to assess moisture content unless otherwise stated.

#### 4.4.3. Storage

For storage in the seed bank at Meise Botanic Gardens, Belgium, seeds were further dried for four months at 15%RH and 20 °C, and then placed in cold storage at 15%RH and −20 °C sealed in aluminium envelopes. Seeds were in cold storage for two months prior to viability evaluation. The moisture content of seeds was taken prior to transfer to cold storage.

### 4.5. Viability Evaluation of Seeds Stored in the Seed Bank

We used embryo rescue techniques to evaluate viability [61,62,63]. This is the most effective measure of *Musa* seed viability compared to whole seed germination [61,62,96] and the tetrazolium chloride test [101] (Simon Kallow, pers. obs.).

We evaluated the viability of batch 1 seed accessions that had been stored in the Meise Botanic Gardens cold storage for two months. No pre-storage viability evaluation had been made. The MC of a subset of three accessions was assessed on removal from storage.

For embryo rescue, seeds were sterilised by soaking them in 96% ethanol for 3 min, followed by 20 min in 1% NaOCl (diluted commercial bleach 5%), containing 1 drop of detergent per 100 mL. Seeds were then rinsed three times in sterile water. Embryos were extracted from seeds using a sterile forceps and scalpel by making an incision in the seed coat next to the micropyle with the scalpel and by manipulating the seed with scalpel and forceps until the testa split open exposing the endosperm and embryo; embryos were then removed by careful manipulation. Embryos were transferred onto autoclaved half MS medium [102] in tubes with the haustorium in contact with medium and the embryonic axis upwards. All procedures were carried out in a laminar flow cabinet. Tubes containing embryos were incubated in the dark at 27 °C for 14 days, after which they were put in a growth chamber for an additional 14 days (24 h photo-period, 27 °C, 50 μE m^−2^ s^−1^ illumination provided by 36 W Osram cool-white fluorescent tubes). Six possible observations were recorded after 28 days: empty (no embryo, identified during excision), contamination, no reaction, callus formation, darkening and germination. *Musa* embryos are regarded as non-dormant when cultured in vitro [61,62,63,96,103], so seeds showing no reaction during this period were considered dead. Embryos that form calluses or that darken are considered alive but unlikely to regenerate into seedlings. An average of 23 ± 10 seeds from an average of 3 hands were tested for 29 accessions. Seed availability for this evaluation was highly constrained. 

### 4.6. Effect of Desiccation

Following the results of the viability evaluation of stored seeds (Section 2.1), batch 2 was collected. We assessed the effect of desiccation on the eight accessions included, using embryo rescue (as previously described). This was done before desiccation and then after seven days of enforced desiccation. Seeds were desiccated by placing them on plastic boats suspended over silica gel sealed in a desiccator. The environment in the desiccator was approximately 2.4% RH and 20 °C. Ten seeds per accession were used both before and after extraction. Moisture content was measured for each accession before and after desiccation using ten seeds.

### 4.7. Prediction of Seed Storage Behaviour 

Seed mass and initial moisture content was used to predict seed storage behaviour according to the model of Hong and Ellis [65] and Ellis et al. [66]. For this, seeds with a 1000 seed weight of less than 2500 g and a moisture content of less than 22% are predicted to have orthodox storage behaviour. Seeds with higher mass and moisture content (>4000 g and >40%MC) are predicted to be recalcitrant. Seeds in-between these limits are predicted to be intermediate. Only seeds in batch 2 were used for this prediction as fresh seed moisture content is a requirement. Seeds were weighed within seven days of extraction whilst maintained in ambient conditions (60–80%RH and 20 °C) to remove excess moisture gained during extraction. Five replicates of 50 seeds were weighed and the mean of this was used to calculate 1000 seed weight for each accession. Moisture content was measured as described above. Moisture content and mass for each accession was then plotted in a scatter chart.

### 4.8. Survival during Dry Storage 

#### 4.8.1. Effect of Maturity 

Two *M. acuminata* subsp. *banksii* accessions from batch 1 were selected from a seemingly more mature (accession #11) and a less mature bunch (accession #4). Maturity level was identified during collecting. The seemingly mature bunch had darker fruit colour and softer pulp texture, as well as more powdery endosperm compared to the less mature bunch (Figure 5). Subsample seeds were selected across all hands and mixed, so that the seeds used reflected the entire accession. For these selected accessions, embryo rescue was carried out after extraction as described above, and then after 1 week, 1, 3 and 6 months of dry storage in a paper bag stored in a dry room (15%RH, 20 °C). Forty eight seeds were used from each accession at each time point. The MC of seeds of each accession at each time point described was measured. 

#### 4.8.2. Effect on Morphology 

Seeds from the selected *M. acuminata* subsp. *banksii* accessions (#4 and #11) were dissected and photographed at the same time points and conditions described above. A digital microscope (Keyence VHX5000) was used at 150–200 x magnification. Ten seeds were used per accession, condition and time point.

### 4.9. Statistical Analysis

Counts from the categorised outcomes of the embryo rescue tests were transformed into lists where each embryo’s reaction was a nominal outcome. These data were then used to build multinomial logistic regression (MLR) models to analyse the log-odds of the embryo rescue outcome category using the *nnet* R package [104]. Maximum models were reduced by comparing Akaike information criterion (AIC) and carrying out the likelihood ratio test. Effects plots were produced from the models by predicting and then plotting data using the effects R package [105]. Statistics were carried out in R v 3.6.2 [106].

## 5. Conclusions

The aim of this study was to assess the viability of banana seeds collected during two collecting missions in order to inform ex situ conservation of banana CWRs. (1) We found that in general *Musa* seeds collected in PNG and stored in the seed bank had low viability. (2) There was considerable variation between accessions, *Musa balbisiana* seeds had significantly higher post-storage viability than other species. (3) Variation within accessions, according to the position in the infructescence, was significant, with seeds of *M. acuminata* subsp. *banksii* from the basal end having lower viability after storage than from the male bud end. (4) Freshly extracted seeds lost much of their viability during desiccation. (5) Predictions of seed storage behaviour based on physical properties indicate that *Musa* seeds are at the threshold of orthodox and intermediate classification; this is in keeping with our embryo rescue results. (6) *M. acuminata* subsp. *banksii* seeds, identified in the field as more mature, had higher viability before and during dry storage than less mature seeds, but this level was also reduced after dry storage. 

This assessment of seed viability demonstrates the importance of advancing understanding of the seed storage behaviour of CWRs. In particular, we show how the ecology and adaption of species and the development of their seeds in time effects the viability of seeds collected for storage. 

## Figures and Tables

**Table 1:** Details of seed collections.

Batch	Accession	Species	Province	Latitude	Longitude	Date Collected
1	1	*M. balbisiana*	Morobe	S 07°03′23″	E 146°34′56″	14/05/2019
1	2	*M. balbisiana*	Morobe	S 07°07′35″	E 146°36′57″	14/05/2019
1	3	*M. balbisiana*	Madang	S 04°41′49″	E 145°36′49″	17/05/2019
1	4	*M. acuminata* subsp*.banksii*	Morobe	S 06°43′34″	E 146°42′40″	14/05/2019
1	5	*M. acuminata* subsp*.banksii*	Morobe	S 06°43′34″	E 146°42′40″	14/05/2019
1	6	*M. acuminata* subsp*.banksii*	Morobe	S 07°13′42″	E 146°36′32″	14/05/2019
1	7	*M. acuminata* subsp*.banksii*	Morobe	S 07°13′42″	E 146°36′32″	14/05/2019
1	8	*M. acuminata* subsp*.banksii*	Morobe	S 06°47′14″	E 146°47′00″	15/05/2019
1	9	*M. acuminata* subsp*.banksii*	Morobe	S 06°46′04″	E 146°46′46″	15/05/2019
1	10	*M. acuminata* subsp*.banksii*	Madang	S 04°41′31″	E 145°36′60″	17/05/2019
1	11	*M. acuminata* subsp*.banksii*	Sandaun	S 02°43′58″	E 141°15′20″	20/05/2019
1	12	*M. acuminata* subsp*.banksii*	Sandaun	S 03°09′53″	E 141°21′57″	21/05/2019
1	13	*M. acuminata* subsp*.banksii*	Sandaun	S 03°09′51″	E 141°18′18″	21/05/2019
1	14	*M. acuminata* subsp*.banksii*	Sandaun	S 02°55′56″	E 141°25′09″	21/05/2019
1	15	*M. acuminata* subsp*.banksii*	Sandaun	S 02°42′17″	E 141°05′36″	22/05/2019
1	16	*M. acuminata* subsp*.banksii*	Sandaun	S 02°42′46″	E 141°05′45″	22/05/2019
1	17	*M. boman*	Sandaun	S 03°01′46″	E 141°19′18″	21/05/2019
1	18	*M. boman*	Sandaun	S 02°48′36″	E 141°23′44″	21/05/2019
1	19	*M. boman*	Sandaun	S 03°09′38″	E 141°02′22″	21/05/2019
1	20	*M. ingens*	Morobe	S 06°48′03″	E 146°46′24″	15/05/2019
1	21	*M. ingens*	Morobe	S 06°47′14″	E 146°47′00″	15/05/2019
1	22	*M. lolodensis*	Sandaun	S 03°01′46″	E 141°19′18″	21/05/2019
1	23	*M. peekelii*	Madang	S 05°09′44″	E 145°44′51″	16/05/2019
1	24	*M. peekelii*	Madang	S 04°55′07″	E 145°45′51″	18/05/2019
1	25	*M. peekelii*	Madang	S 04°55′07″	E 145°45′51″	18/05/2019
1	26	*M. schizocarpa*	Madang	S 04°44′17″	E 145°38′60″	17/05/2019
1	27	*M. schizocarpa*	Sandaun	S 02°44′19″	E 141°21′11″	19/05/2019
1	28	*M. schizocarpa*	Sandaun	S 02°43′58″	E 141°15′20″	20/05/2019
1	29	*M. schizocarpa*	Sandaun	S 02°37′02″	E 141°00′52″	22/05/2019
2	30	*M. acuminata* subsp*.banksii*	Madang	S 05°11′58″	E 145°39′30″	16/10/2019
2	31	*M. acuminata* subsp*.banksii*	Madang	S 05°11′58″	E 145°39′30″	16/10/2019
2	32	*M. maclayi*	West New Britain	S 05°33′54″	E 150°47′29″	03/10/2019
2	33	*M. maclayi*	West New Britain	S 05°33′54″	E 150°47′29″	03/10/2019
2	34	*M. maclayi*	West New Britain	S 05°33′54″	E 150°47′29″	03/10/2019
2	35	*M. maclayi*	West New Britain	S 05°33′54″	E 150°47′29″	03/10/2019
2	36	*M. schizocarpa*	Madang	S 05°11′58″	E 145°39′30″	16/10/2019
2	37	*M. balbisiana*	Madang	S 05°11′58″	E 145°39′30″	15/10/2018

## Supplementary Materials

The following are available online at https://www.mdpi.com/2223-7747/9/9/1243/s1, Figure S1: (A) Annual precipitation, and (B) precipitation of the driest quarter (three-month period), across the native distribution of the species evaluated in this study. Data extracted from WorldClim v2.0 based on occurrence records of species (data compiled by A. Mertens). Green dots displays means. Figure S2: Batch 1 fruits during processing after arrival at Meise Botanic Gardens, Belgium. Fruits are separated according to hand position on the bunch. Table S1: Multinomial logistic regression coefficients in log-odds (logits) and standard deviations in parentheses. (A) Embryo rescue outcome of batch 1 post storage. (B) Embryo rescue outcome of post-storage *Musa acuminata* subsp. *banksii* accessions in batch 1 according to hand position (1 being at the basal peduncle end of the infructescence). (C) Embryo rescue outcome of seeds from batch 2 after drying in a desiccator for seven days, a factor with two levels (‘Wet’ and ‘Dry’). Embryo rescue outcome categorised after 28 days. Stars designate significance levels for *p* values * ≤ 0.05, ** ≤ 0.01, *** ≤ 0.001.
