# Supplementary material for: Challenges for Ex Situ Conservation of Wild Bananas: Seeds Collected in Papua New Guinea Have Variable Levels of Desiccation Tolerance
Source: Plants (Basel). 2020 Sep 21;9(9):1243. doi: 10.3390/plants9091243 (PMC7570212; doi:10.3390/plants9091243)
Supplement: Supplementary file 1 [file plants-09-01243-s001.pdf]

Supplementary Figures

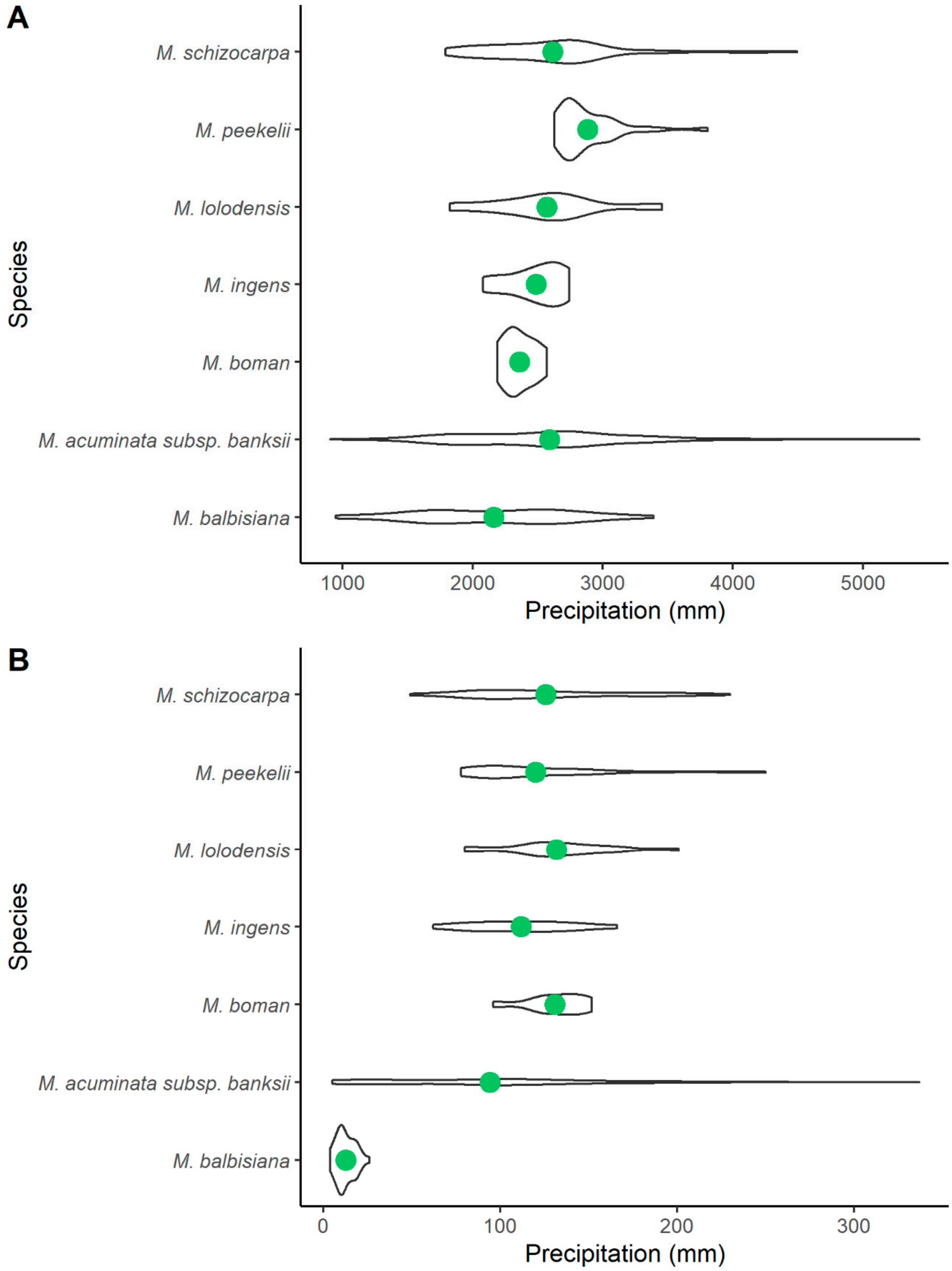

Figure S1. (A) Annual precipitation, and (B) precipitation of the driest quarter (three-month period), across the native distribution of the species evaluated in this study. Data extracted from WorldClim v2.0 based on occurrence records of species (data compiled by A. Mertens). Green dots displays means.

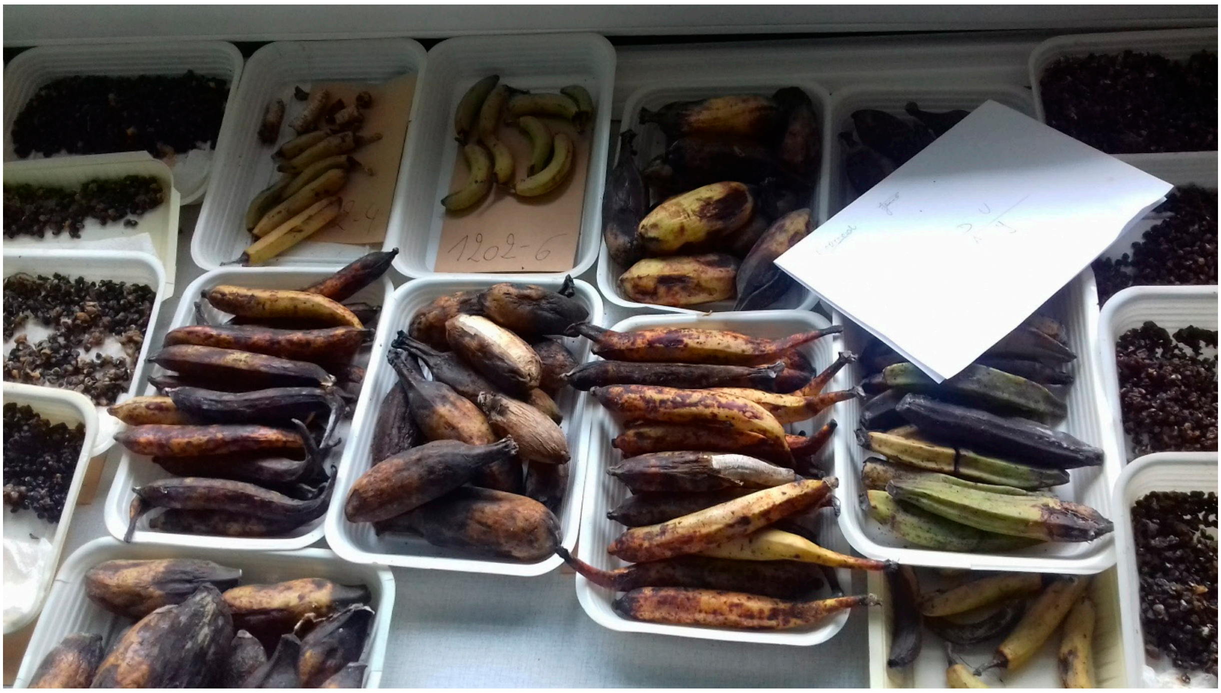

Figure S2. Batch 1 fruits during processing after arrival at Meise Botanic Gardens, Belgium. Fruits are separated according to hand position on the bunch.

Supplementary Tables

Table S1. Multinomial logistic regression coefficients in log-odds (logits) and standard deviations in parentheses. (A) Embryo rescue outcome of batch 1 post storage. (B) Embryo rescue outcome of post-storage *Musa acuminata* subsp. *banksii* accessions in batch 1 according to hand position (1 being at the basal peduncle end of the infructescence). (C) Embryo rescue outcome of seeds from batch 2 after drying in a desiccator for seven days, a factor with two levels ('Wet' and 'Dry'). Embryo rescue outcome categorised after 28 days. Stars designate significance levels for p values *<=0.05, **<=0.01, ***<=0.001.

A

	Embryo rescue category				
Species	empty	contamination	callus	darkening	germination
M. balbisiana (constant)	-1.163*	-2.774**	-24.944***	-15.240***	0.865**
	(0.512)	(1.031)	(0.251)	(0.237)	(0.298)
M. acuminata subsp. *banksii*	0.553	-0.762	19.575***	10.094***	-2.311***
	(0.516)	(1.051)	(0.251)	(0.436)	(0.308)
M. boman	-0.069	-20.419***	-7.078***	14.008***	-3.350***
	(0.669)	(0.000)	(0.000)	(0.424)	(0.794)
M. ingens	0.134	-17.121***	-5.176***	-6.141***	-14.748
	(0.731)	(0.000)	(0.000)	(0.000)	(276.434)
M. lolodensis	-14.937***	-16.006***	-3.587***	13.294***	-2.118*
	(0.00001)	(0.000)	(0.000)	(0.905)	(0.855)
M. peekelii	-0.661	-19.908***	-7.656	12.904***	-4.299***
	(0.703)	(0.000)	(0.000)	(0.548)	(1.059)

M. schizocarpa	-15.198*** (0.00005)	-0.558 (1.449)	-8.257*** (0.000)	14.210*** (0.383)	-1.799*** (0.464)

B

	Embryo rescue category				
Variable	empty	contamination	callus	darkening	germination
Hand position	0.026	-0.209*	-0.215	0.214	0.110***
	(0.020)	(0.090)	(0.223)	(0.143)	(0.026)
Intercept	-0.758***	-2.507***	-4.315***	-6.556***	-2.117***
	(0.130)	(0.436)	(1.075)	(1.175)	(0.185)

C

	Embryo rescue category				
Variable	empty	contamination	callus	darkening	germination
Condition 'Wet'	-6.698	3.970**	2.179	3.460***	3.747***
	(69.103)	(1.298)	(1.361)	(0.814)	(0.627)
Constant 'Dry'	-2.872***	-3.970***	-3.277***	-2.518***	-0.415
	(0.593)	(1.009)	(0.720)	(0.465)	(0.218)
